# Multiobjective Decision Making Policies and Coordination Mechanisms in Hierarchical Organizations: Results of an Agent-Based Simulation

**DOI:** 10.1155/2014/875146

**Published:** 2014-07-24

**Authors:** Stephan Leitner, Friederike Wall

**Affiliations:** Department of Controlling and Strategic Management, Faculty for Business and Economics, Alpen-Adria Universität, 9020 Klagenfurt, Austria

## Abstract

This paper analyses how different coordination modes and different multiobjective decision making approaches interfere with each other in hierarchical organizations. The investigation is based on an agent-based simulation. We apply a modified NK-model in which we map multiobjective decision making as adaptive walk on multiple performance landscapes, whereby each landscape represents one objective. We find that the impact of the coordination mode on the performance and the speed of performance improvement is critically affected by the selected multiobjective decision making approach. In certain setups, the performances achieved with the more complex multiobjective decision making approaches turn out to be less sensitive to the coordination mode than the performances achieved with the less complex multiobjective decision making approaches. Furthermore, we present results on the impact of the nature of interactions among decisions on the achieved performance in multiobjective setups. Our results give guidance on how to control the performance contribution of objectives to overall performance and answer the question how effective certain multiobjective decision making approaches perform under certain circumstances (coordination mode and interdependencies among decisions).

## 1. Introduction and Research Question

During the last decades, changes in environmental conditions have brought organizations to revise their management approaches. In fact, today the major challenges are increased complexity and the need to consider multiple and potentially conflicting objectives in corporate decision making, instead of focusing mainly on one goal as encapsulated in the value-based management approach (e.g., [1]). For these developments, there are several lines of explanation. First, the rapid technological change and the growing globalization increase the complexity and turbulence of the organizations' environment and lead to competition being intensified [2]. Second, the consideration of different stakeholder interests in decision making has become critical to the organizations' success [3]. Third, the call for sustainability claims to balance economic, ecologic, and social objectives [4].

These developments evoke an increasing complexity which organizations have to face [5]. In particular, it is likely that there are more complex interdependencies among decisions and the organizations' target systems become more complex, too. Due to these more complex interactions and the multiplicity of objectives to be pursued simultaneously, the coordination within organizations becomes more difficult. Adaptions in the respective organizations' design might be one option to face these challenges.

Although the methods of multiobjective decision making are widely investigated for the case of individuals (e.g., [6]), they are rarely researched for hierarchical organizations. In particular for the case of distributed decision making authority, the modes of multiobjective decision making interfere with varying coordination mechanisms, whereby the respective impact on the achieved performance is widely unclear. In addition, decisions made in the own best interest by the decentral decision makers (i.e., opportunistically made decisions) might have an impact on the efficiency of the multiobjective decision making mode in combination with certain coordination mechanisms. In this paper, we particularly focus on this question. We investigate whether and how different coordination mechanisms and different multiobjective decision making policies interfere with each other and how they affect the achieved organizational performance. In our research, we do not seek to optimize search algorithms for alternative strategies. We do rather analyze how performance in hierarchical setups evolves in case of certain coordination modes and different multiobjective decision making methods, which are widespread to organizational practice. We intend to derive findings on the following questions: “How sensitive is the achieved performance to (1) the selection of certain decision making modes and to (2) the selection of certain coordination mechanisms in setups with different natures of interdependencies among decisions?” The aim of this paper is to derive implications for the selection of multiobjective decision making approaches and the simultaneous selection of coordination mechanisms in certain setups of interdependencies among decisions with respect to maximizing the organizations' performance.

In order to investigate the research question, we employ an agent-based simulation approach which allows for mapping hierarchical organizations, different modes of coordination, interacting agents, and different methods of multiobjective decision making. Furthermore, simulation experiments allow for investigating the macrolevel outcome (i.e., the organizational performance) that results from microlevel interactions (the decentral decision makers' decisions and the interactions among these decisions) [7]. Controlling the multitude of issues and disentangling effects of variables under research from other effects would find the boundaries of empirical research [8]. Due to this potential complexity and unpredictability of repeated simple patterns, formal modeling might lead to intractable dimensions [9]. Agent-based simulation, on the contrary, appears to be a powerful research method to face the complexity of the research problem.

In particular, the mapped organizations consist of a number of decision makers and pursue two objectives. We simulate variations in the multiobjective decision making approach and variations in the coordination mode and investigate the impact of the two variables on the achieved performances over time. In addition, we vary the nature of interdependencies among the partial (decentral) decision making problems. For each objective, we consider interdependencies among the departmental decision problems but not between objectives. Thus, the achieved organizational performance is inextricably linked to the respective decisions and, of course, also to the interactions among the decisions [8, 10–12]. In addition, if simple patters of interaction are repeated multiple times they potentially lead to unpredictable outcomes [13]. This is why we employ agent-based simulation.

## 2. Simulation Model

In order to investigate the research questions, we set up a computational model on the basis of the NK-model as introduced by Kauffman et al. [14–17]. In order to build the simulator we use Visual Basic for Applications. After its introduction into the field of management by Levinthal [18], the model has been applied to several contexts, for example, imitation strategies [19, 20], organizational design, turbulence, and complexity [21–23], multiple performance goals [24], innovation [7, 25], manufacturing [26], and situated learning [27]. As stated by Ethiraj and Levinthal [24], the wide acceptance of the NK-model among scholars increases the confidence in its properties.

Furthermore, we have decided for the NK-model because it is explicitly designed to investigate interactions among its components (which, in our case, are departmental decisions). Based on the basic NK-model [14–17] and in consideration of earlier investigations by Ethiraj and Levinthal [24], our model applies simultaneous adaptive walks on multiple performance landscapes. In our model, each managerial decision affects the performance on multiple objectives whereby each objective is represented by one performance landscape.

Since the NK-model has been widely applied in management research; several extensions have evolved. With respect to cognitive representations of the decision problem and vicarious learning, Gavetti [28] and Gavetti and Levinthal [29] map search processes that differ from the basic NK-model. Altenberg [30] and Frenken [31] apply a search space that changes over time; that is, contrary to the basic NK-model, the number of decisions and interdependencies among them are modeled as an endogenous variable. The NK-model assumes interdependencies among decisions to be randomly distributed. Ethiraj and Levinthal [32], Ethiraj et al. [19] and Rivkin and Siggelkow [21, 22] relax this assumption and analyze effects of modularity and patterned interactions among decisions.

We apply the model to setups in which organizations pursue multiple objectives simultaneously. In particular, each performance landscape represents one objective, as originally considered in the NK-model, but due to multiple objectives, each decision affects performances achieved with respect to multiple objectives meaning that multiple performance landscapes are incorporated into our simulation model. In order to give a comprehensive overview of the model's structure, three main features have to be discussed in detail: (1) the design of hierarchical organizations, (2) the representation of the performance landscapes, and (3) the mapped methods of multiobjective decision making.

### 2.1. The Hierarchical Design

We map organizations as systems of interdependent choices [33]; that is, we conceptualize organizations to search along a multidimensional decision space for optimal configurations rather than making decisions in a single-dimensional setup [20]. The decision problems which the mapped organizations face are represented by the respective performance landscapes. In particular, each landscape represents one objective and—depending on the mode of multiobjective decision making—organizations seek to increase performance across performance landscapes. The number of decisions and the architecture of performance landscapes are constant along the observation period.

The mapped organizations face a ten-dimensional binary decision problem; that is, in each period *t* ∈ {1,…, *T*} agents make decisions *n* ^*i*,*t*^ ∈ *N* with *n* ^*i*,*t*^ ∈ {0,1} and *i* ∈ {1,…, |*N*|}. Due to the binarity of the options for each single decision, there are 2^|*N*|^ different configurations for the overall decision problem expressed by vectors *C* = (*n* ^*i*=1^,…, *n* ^*i*=|*N*|^). The configuration of decisions for period *t* is denoted as *C* ^*t*^ = (*n* ^*i*=1,*t*^,…, *n* ^*i*=|*N*|,*t*^). The starting configuration *C* ^*t*=0^ is selected randomly.

The decisions *n* ^*i*,*t*^ affect the performance of all objectives *g* ∈ *G*. In each period *t* and for each objective *g*, the decisions *n* ^*i*,*t*^ make a contribution *p*_*g*_^*i*,*t*^ to the overall performance per objective *P*_*g*_^*t*^. Due to the interdependencies among decisions, the performance contribution *p*_*g*_^*i*,*t*^ additionally to decision *n* ^*i*,*t*^ may be affected by *K*_*g*_^*i*^ other decisions *n*_*k*_^*j*,*t*^ whereby *i*, *j* ∈ {1,…, |*N*|}, *k* ∈ {1,…, *K*_*g*_^*i*^}, and *i* ≠ *j*. For each period *t* and each performance contribution *p*_*g*_^*i*,*t*^, the payoff-function *f*_*g*_^*i*^ randomly draws a value from the uniform distribution *U*[0,1]; that is

(1) $$\begin{array}{c} p_{g}^{i,t}=f_{g}^{i}\left(n^{i,t};n_{k=1}^{j,t},\ldots ,n_{k=K_{g}^{i}}^{j,t}\right), \end{array}$$

whereby *i*, *j* ∈ {1,…, |*N*|}, *i* ≠ *j*, and 0 ≤ *p*_*g*_^*i*,*t*^ ≤ 1. Whenever any of the coupled decisions changes, the value for *p*_*g*_^*i*,*t*^ is redrawn from the underlying distribution. We map all performance contributions *p*_*g*_^*i*,*t*^ to contribute to the performance per objective equally. Thus, the overall performance *P*_*g*_^*t*^ results in the normalized sum of performance contributions *p*_*g*_^*i*,*t*^; that is,

(2) $$\begin{array}{c} P_{g}^{t}=\frac{1}{\left|N\right|}\overset{\left|N\right|}{\underset{i=1}{\sum }}p_{g}^{i,t}. \end{array}$$

**Figure 1:**
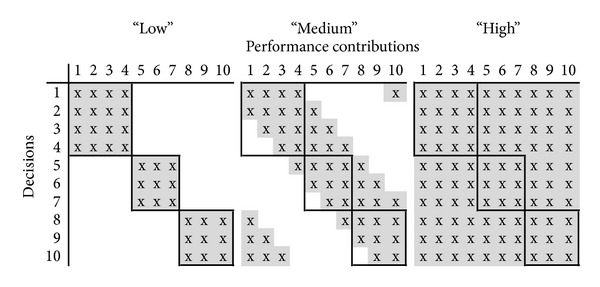
Interdependence matrices.

Our organizations consist of decentralized units *d* ∈ *D* and one central unit *h*. With respect to prior research on multiobjective decision making based on the NK-model (e.g., [24]), the mapping of hierarchical structures is a novelty. In particular, we map organizations that consist of three decentral units whereby two units are in scope of three decisions and one unit is in scope of four decisions (cf. also Figure 1; the solid lines represent the decentral structure). For each decentral unit *d* we denote the set of decisions within their own area of responsibility as *N* ^own_*d*_^. From the perspective of unit *d*, the decisions delegated to the* other* units are denoted as *N* ^res_*d*_^.

We map decentral decision makers as agents that seek to enhance their individual utility via incremental changes whereby the characterizations of the utility functions are dependent on the method of multiobjective decision making (for the respective utility functions cf.  Section 2.3). Efforts for stepwise improvement go along with literature on organizational learning [34] and prior modeling efforts (e.g., [22]) while the agents' selfishness is consistent with the economic literature [35]. Due to bounded rationality [36], agents do not envision all possible alternative configurations of departmental decisions *N* ^own_*d*_^. Rather, they randomly discover two alternative configurations of decisions that differ in one, respectively, two partial decisions *n* ^*i*,*t*^ from the status quo.

Along with the status quo, in each period the decentral units *d* evaluate three alternative configurations of decisions. The decentral units *d* rank two of the alternative configurations under evaluation, on the basis of which alternative promises the highest improvement in individual utility. Depending on the limitation of proposals, one alternative (i.e., that configuration of decisions that promises the least performance) is not considered in the order of preferences. The ranking of departmental decisions *N* ^own_*d*_^ is denoted as vectors *V*_*r*_^own_*d*_,*t*^ with *r* = {1,2} indexing the assigned rank.

We analyze the impact of organizational design options (i.e., the selection of the multiobjective decision making approach and the selection of the coordination mode) on overall performance, that is, the performances achieved at the organizational level with respect to the multiple objectives pursued.

The mode of coordination is one of the major organizational design options [37]. The coordination mode determines how the overall configuration of decisions for the following period *t* + 1 is built. In our model, we consider two different coordination modes: (1) fully decentralized coordination and (2) a central mode of coordination. In case of* full decentralization*, the decentral units decide and act autonomously in their areas of responsibility *N* ^own_*d*_^ [38]. In period *t* + 1, the overall configuration of decisions is given by a concatenation of the departmental decision vectors ranked first. In case of the* central mode of coordination*, each decentral unit is eligible to send two proposals *V*_*i*_^own_*d*_,*t*^ to the central unit. All proposals are evaluated on the basis of the central unit's utility function (cf. equation (6)). The central unit evaluates concatenations of all proposals *V*_*i*_^own_*d*_,*t*^ and residual decisions according to the status quo *D* ^res_*d*_,*t*^ and selects that proposal, that promises the highest performance, to be the configuration implemented in the following period *t* + 1. The notion of overall performance is dependent on the selection of the multiobjective decision making mode (cf.  Section 2.3.4).

One further design element is the incentive scheme. The incentive scheme is reflected in the subunits' utility functions (cf.  Section 2.3) and, hence, directly affects the outcome of the ranking of alternatives. We consider a linear incentive scheme in which for every period *t* the decentral units *d* are rewarded on the basis of the performance *P*_*g*_^*t*^ of each objective *g*. In particular, the basis for the calculation of the individual compensation depends on performance of intraunit decisions *N*_*g*_^own_*d*_,*t*^ and on the performance of the residual decisions *N*_*g*_^res_*d*_,*t*^ with different weights denoted as *w*_*g*_^own_*d*_^ and *w*_*g*_^res_*d*_^, respectively.

### 2.2. Representation of the Performance Landscapes

The complexity in hierarchical organizations critically depends on the nature of interdependencies among decisions [39]. We regard complexity to be a function of the choice of organizational design options and the organizational environment. On the one hand, interdependencies among decisions are dictated by the decision problem itself [23]. On the other hand, organizations can face this given complexity in considering interdependencies among decisions in the structure of decentralized units. Hence, building units or assigning decision making authority with respect to interdependencies among decisions might crucially affect the achieved performances.

According to Section 2.1, we describe interdependencies among decisions by parameter *K*_*g*_^*i*^. Increasing interdependencies *K*_*g*_^*i*^ lead to performance landscapes to be more rugged [16]. With respect to the search strategy mapped (i.e., incremental improvement), a lower level of interdependencies leads to more starting configurations of decision *C* ^*t*=0^ to be in the basin of attraction of the global maximum. Higher levels of interdependencies *K*_*g*_^*i*^ lead to a higher number of local maxima [40]; that is, the configurations of decisions on the basis of which the performance cannot be further improved by local search algorithms. Once an organization reaches such a trap, the status quo of the configuration of decisions is likely to be constant for the remaining observation period [24].

We follow the basic NK-framework [15–17] and use interdependence matrices in order to represent functional dependencies among decisions (for convenience the superscript *t* is suppressed in the discussion of the natures of interdependencies). Due to the |*N*|-dimensionality of the decision problem, the matrices *M* are of size |*N* | ∗ | *N*|. The set of decisions *N* is assigned to the vertical axis while the horizontal axis represents the payoff functions *f*_*g*_^*i*^ (cf. Figure 1). An “*x*” in cell *m*_*ij*_ with *i*, *j* ∈ {1,…, |*N*|} and *i* ≠ *j* indicates that additionally to the performance contribution *p*_*g*_^*i*^, the decision *n* ^*i*^ affects the performance contribution *p*_*g*_^*j*^. Consequentially, empty cells *m*_*ij*_ indicate that there is no functional dependency among the decision *n* ^*i*^ and the performance contribution *p*_*g*_^*j*^ (cf. equation (1)). In our mapping, the performance contribution *p*_*g*_^*i*^ is functionally dependent on the decision *n* ^*i*^ in all cases. Thus, the entries on the main diagonal are always set to “*x*.”

### 2.3. Methods of Multiobjective Decision Making

In this simulation study, we investigate three different methods of multiobjective decision making. In particular, we map the method of (1) equal weighting according to which all objectives are pursued with the same importance. Furthermore, our analysis captures (2) satisficing and (3) schism approaches [41]. In the case of satisficing approaches, aspiration levels for certain objectives are stated, while in the case of schism approaches, the preferences for objectives change over time. The methods of multiobjective decision making affect the departments' and the central unit's utility functions. The next sections outline the methods of multiobjective decision making under investigation and give the respective departments' utility functions.  Section 2.3.4 discusses the headquarters' utility functions.

#### 2.3.1. Equal Weighting

In case of (1)* equal weighting*, the decision makers do not have to articulate preferences for single objectives; they rather decide for all objectives to be pursued with the same importance [42, 43]. Consequently, with respect to multiple objectives and the linear incentive scheme (as stated in Section 2.1), departments are compensated on the basis of their performance, that is, the weighted sum of departmental and residual performance achieved with all objectives. We formalize the departments' utility function in the case of equal weighting by

(3) $$\begin{array}{c} U_{d}^{t}=\overset{\left|G\right|}{\underset{g=1}{\sum }}\left(w_{g}^{\text{ow}{\text{n}}_{d}}\frac{\sum_{i\in N^{\text{ow}{\text{n}}_{d}}}^{}p_{g}^{i,t}}{\left|N^{\text{ow}{\text{n}}_{d}}\right|}+w_{g}^{\text{re}{\text{s}}_{d}}\frac{\sum_{i\in N^{\text{re}{\text{s}}_{d}}}^{}p_{g}^{i,t}}{\left|N^{\text{re}{\text{s}}_{d}}\right|}\right). \end{array}$$

#### 2.3.2. Satisficing Approach

In order to operationalize the (2)* satisficing approach*, we introduce aspiration levels *s*_*g*_ ∈ [0,1] which are constant along the entire observation period. The function *f* ^*g*^(*s*_*g*_) = *g* defines the objective *g* for which the aspiration level *s*_*g*_ is stated. Decision makers seek to, at least, satisfy the aspiration level before they consider the remaining objectives in the evaluation of alternatives (cf. Section 2.1). The period in which the aspiration level is achieved or exceeded is denoted as *t* ^*s*_*g*_^. In the case of satisficing approaches, for the periods *t* ≤ *t* ^*s*_*g*_^, the units solely pursue one objective, that is, objective *f* ^*g*^(*s*_*g*_) = *g*. Once the performance *P*_*f*^*g*^(*s*_*g*_)_^*t*^ exceeds the stated aspiration level, no alternative configuration of decisions will be realized that leads to the respective performance *P*_*f*^*g*^(*s*_*g*_)_^*t*^ falling below the aspiration level. Similar to (1), in case of equal weighting, for periods *t* > *t* ^*s*_*g*_^, organizations assign equal importance to all objectives. The corresponding utility functions results in

(4) $$\begin{array}{c} U_{d}^{t}=\{\begin{array}{cc} w_{f^{g}\left(s_{g}\right)}^{\text{ow}{\text{n}}_{d}}\frac{\sum_{i\in N^{\text{ow}{\text{n}}_{d}}}^{}p_{f^{g}\left(s_{g}\right)}^{i,t}}{\left|N^{\text{ow}{\text{n}}_{d}}\right|}+w_{f^{g}\left(s_{g}\right)}^{\text{re}{\text{s}}_{d}}\frac{\sum_{i\in N^{\text{re}{\text{s}}_{d}}}^{}p_{f^{g}\left(s_{g}\right)}^{i,t}}{\left|N^{\text{re}{\text{s}}_{d}}\right|} & \\ \;\forall t\leq t^{s_{g}} & \\ \overset{\left|G\right|}{\underset{g=1}{\sum }}\left(w_{g}^{\text{ow}{\text{n}}_{d}}\frac{\sum_{i\in N^{\text{ow}{\text{n}}_{d}}}^{}p_{g}^{i,t}}{\left|N^{\text{ow}{\text{n}}_{d}}\right|}+w_{g}^{\text{re}{\text{s}}_{d}}\frac{\sum_{i\in N^{\text{re}{\text{s}}_{d}}}^{}p_{g}^{i,t}}{\left|N^{\text{re}{\text{s}}_{d}}\right|}\right) & \\ \;\forall t>t^{s_{g}}. & \end{array} \end{array}$$

#### 2.3.3. Schism Approach

Schism approaches correspond to the concept of temporal separation [34]. We map decision makers to pursue only a subset of objectives *G* at the same time. Furthermore, we map two characterizations of schism approaches, that is, (1) short-run schism and (2) long-run schism. In case of short-run schism, the volatility in the preference for certain objectives is higher than it is for the case of long-run schism. In particular, in the case of short-run schism, preferences for objectives change every period while in the case of long-run schism, preferences change every ten periods (cf. also [43]).

In order to operationalize the schism approaches, we introduce weighting factors *q*_*g*_^*t*^ ∈ {0,1} which are exogenously given. These factors represent the preferences for objectives from the central unit's point of view. As outlined above, in the case of short-run schism the preferences change every period, that is, for  all  *q*_*g*_^*t*^ : *q*_*g*_^*t*^ ≠ *q*_*j*_^*t*^ and *q*_*g*_^*t*+1^ ≠ *q*_*g*_^*t*^, while in the case of long-run schism the preferences are constant for ten periods, that is, for  all  *q*_*g*_^*t*^ : *q*_*g*_^*t*^ ≠ *q*_*j*_^*t*^ and *q*_*g*_^*t*+10^ ≠ *q*_*g*_^*t*^, where *g*, *j* ∈ *G* and *g* ≠ *j*. If schism approaches are applied, the decentral units' utility function results in

(5) $$\begin{array}{c} U_{d}^{t}=\overset{\left|G\right|}{\underset{g=1}{\sum }}\left[q_{g}^{t}\left(w_{g}^{\text{ow}{\text{n}}_{d}}\frac{\sum_{i\in N^{\text{ow}{\text{n}}_{d}}}^{}p_{g}^{i,t}}{\left|N^{\text{ow}{\text{n}}_{d}}\right|}+w_{g}^{\text{re}{\text{s}}_{d}}\frac{\sum_{i\in N^{\text{re}{\text{s}}_{d}}}^{}p_{g}^{i,t}}{\left|N^{\text{re}{\text{s}}_{d}}\right|}\right)\right]. \end{array}$$

#### 2.3.4. The Central Unit's Utility Function

While decentralized units aim to maximize their own utility functions (cf. equations (3), (4) and (5)), the central unit seeks to maximize overall performance; that is, contrary to the decentral units' utility functions, the central unit does not distinguish between departmental and residual performance. With respect to the methods of multiobjective decision making mapped, the central unit's utility function results in

(6) $$\begin{array}{c} U_{h}^{t}=\{\begin{array}{cc} \frac{1}{\left|G\right|}\overset{\left|G\right|}{\underset{g=1}{\sum }}P_{g}^{t} & \text{if}\;\;\text{equal}\;\;\text{weighting}\\ P_{f^{g}(s_{g})}^{t} & \text{if}\;\;\text{satisficing}\;\;\text{approach},\text{for}\;\;t\leq t^{s_{g}}\\ \frac{1}{\left|G\right|}\overset{\left|G\right|}{\underset{g=1}{\sum }}P_{g}^{t} & \text{if}\;\;\text{satisficing}\;\;\text{approach},\text{for}\;\;t>t^{s_{g}}\\ \frac{1}{\left|G\right|}\overset{\left|G\right|}{\underset{g=1}{\sum }}\left(q_{g}^{t}P_{g}^{t}\right) & \text{if}\;\;\text{schism}\;\;\text{approach}. \end{array} \end{array}$$

In case of equal weighting and satisficing approaches (for periods *t* > *t* ^*s*_*g*_^), the central unit aims to maximize the overall performance considering all objectives simultaneously (whereby in the case of satisficing approaches performance of the objective for which the aspiration level is fixed, must not fall below that level). If aspiration levels are applied, for periods *t* ≤ *t* ^*s*_*g*_^ the central unit solely takes into account that objective the aspiration level is applicable for. In case of schism approaches, preferences for objectives change over time. Depending on these preferences, the central unit seeks to increase the performance of the respective objective (cf. equation (6)).

## 3. Results

In this simulation study each organization is in charge of taking ten decisions and pursuing two objectives simultaneously. Performance is observed for 100 periods. Hence, |*N* | = 10, |*G* | = 2 and *T* = 100. The hierarchical setup of the computational model corresponds to Section 2.1.

We limit our research to three exemplary natures of interdependencies among decisions (for the investigated natures of interdependencies cf. also [42, 43]). In the case* low*, the decisions within a unit are fully interdependent but there is no cross-unit interdependence. Thus, the decisions *N* ^own_*d*_^ do not affect residual performance. For each decentral unit *d*, the *K* values are constant along intraunit decisions; that is, *K*_*g*_^*i*^ = |*N* ^own_*d*_^ | −1 (cf. Figure 1, panel “low”). This pattern of interactions is comparable to the modular setup of organizations as investigated by Rivkin and Siggelkow [22]. With reference to small world networks, the nature of interdependencies* medium* is characterized by a high level of clustering [44]. The interdependencies among decisions are clustered along the main diagonale of the matrix *M*, *K*_*g*_^*i*^ = 4 for all all decisions. This pattern of interactions results in intraunit decisions *N* ^own_*d*_^ being partly interdependent but they are also partly interacting with the other units' decisions. A unit's performance is reciprocally dependent on the other units' decisions but the intraunit decisions also affect the residual performance (cf. Figure 1, panel “medium”). We also map a* high* level of interdependencies in which all decisions are fully interdependent; that is, *K*_*g*_^*i*^ = 9 (cf. Figure 1, panel “high”). For each of the multiple objectives, a distinct performance landscape is generated. Each of these performance landscapes can follow the three natures of interdependencies among decisions as outlinedabove.

For the current investigation, we analyze incentive schemes that put more weight on intraunit performance than on residual performance, which may cause a divergence of interest between the decentral units *d* and the central unit *h*. We set *w*_*g*_^own_*d*_^ = 1 and *w*_*g*_^res_*d*_^ = 0.5. With two objectives and three interdependence structures, six combinations of natures of interdependencies are possible. Furthermore, we analyze two coordination modes and five methods of multiobjective decision making (equal weighting; satisficing approach for objective one and two, respectively; long-run schism; short-run schism). Hence, in sum our simulations cover 60 different parameter settings.

All presented results are based on 450 landscapes per objective whereby for each landscape 20 adaptive walks are simulated. The results for each combination of different levels of interdependencies are based on 9,000 simulation runs. We give two measures for performance. On the one hand, we report achieved performances after 100 periods *P*_*g*_^*t*=100^ as a snapshot of final performance (cf. equation (2)). On the other hand, we report the average performance per objective *g* over the observation period *T* and all 9,000 simulation runs as measure for performance over time *P*_*g*_^avg^; that is,

(7) $$\begin{array}{c} P_{g}^{\text{avg}}=\frac{1}{9,000\cdot T}\overset{9,000}{\underset{m=1}{\sum }}\overset{T}{\underset{t=1}{\sum }}P_{g}^{t,m} \end{array}$$

with *m* indexing the simulation runs. *P*_*g*_^avg^ can also be regarded as a condensed measure of the speed of performance improvement over all 100 periods [23]. Furthermore, the measures for overall performance are given by the averaged performance contributions of all objectives; that is, *P*_all_^*t*=100^ = 1/|*G* | ∑_*g*=1_^|*G*|^ *P*_*g*_^*t*=100^ and *P*_all_^avg^ = 1/|*G* | ∑_*g*=1_^|*G*|^ *P*_*g*_^avg^.

We investigate the effects of the choice of coordination mode on performance and effectivity of multiobjective decision making methods in two steps. First, we analyze the impact of the coordination mode and the nature of interdependencies among decisions on the organizational performance for each multiobjective decision making method separately. This is to answer the question how sensitive the organizational performance achieved with certain multiobjective decision making approaches is to the selection of the coordination mode. In the second step, we analyze the achieved performances across the different multiobjective decision making methods. This allows us to answer the question which decision making mode and which coordination mode appears appropriate for specific natures of interdependencies among decisions.

### 3.1. Equal Weighting

For the case of equal weighting (cf.  Table 1), we find that in the central as well as in the decentral coordination mode, increasing the complexity of the interdependencies among decisions leads to decreasing final performances and also to a decreasing speed of performance improvement. In the majority of the scenarios, only slight differences between the performances achieved with the central and the decentral coordination mode can be observed. In particular, with increasing complexity of the interdependencies among decisions, the decentral coordination mode appears to lead to (slightly) higher performances than the central coordination mode. This is remarkable since increasing complexity potentially increases the probability for negative external effects among decisions. Moreover, in our setting the incentive scheme favors departmental myopia. This holds for the presented final performances as well as for the presented average performances.

Not surprisingly, pursuing objectives with the same natures of interdependencies among decisions leads to very similar levels of achieved final performances and to a very similar speed of performance improvement for the two objectives. If the simulated organizations pursue two objectives with different natures of interdependencies, the results indicate that in the majority of scenarios the higher final and average performance can be achieved for the less complex objective. Only for the scenario* medium/high*, in the central coordination mode a higher final performance and a higher speed of performance improvement can be observed for the more complex objective. For the scenario* low/high*, almost equal performances for the two objectives can be observed in the central coordination mode. Thus, the presented results suggest that with increasing the complexity of interdependencies among decisions, the central coordination mode has a positive effect on the achieved performance with respect to the more complex objective.

### 3.2. Satisficinig Approaches

Similar to the case of equal weighting, in the case of satisficing approaches (cf. Tables 2 and 3) both the final performances and the speed of performance improvement decrease with rising the complexity of interdependencies among decisions. Only slight differences between the performances achieved with the central and the decentral coordination mode can be observed whereby, as in the case of equal weighting, the decentral coordination mode appears to be slightly superior to the central coordination mode. Thus, the results suggest only a slight sensitivity of the achieved performances and the speed of performance improvement to the coordination mode.

For all scenarios, the presented results suggest that the higher level of performance can be achieved for that objective the aspiration level is fixed for. For scenarios in which* objectives with the same natures of interdependencies* are pursued, from the single-objective perspective it makes a difference for which objective the aspiration level is fixed while from the overall-performance perspective this decision does not affect the performance. For scenarios in which two* objectives with different natures of decision interdependencies* are pursued, the presented results indicate that fixing the aspiration level for the objective with the less complex interactions among decisions affects the overall performance positively. This finding also holds for the speed of performance improvement in the central as well as the decentral coordination mode. Thus, our results suggest that, with respect to overall performance and speed of performance improvement, investing resources into the accomplishment of the less complex objective appears to be superior to investing resources into the accomplishment of the more complex objective.

### 3.3. Schism Approaches

As in the cases of the previously analyzed methods of multiobjective decision making, for short-run (cf.  Table 4) as well as for long-run schism approaches (cf.  Table 5) a decrease in the final performances and a decrease in the speed of performance improvement can be observed with increasing the complexity of interactions among decisions. The results suggest a sensitivity of the performance measures to the level of complexity of decision interdependencies for the central as well as for the decentral coordination mode.

In the case of* long-run schism* (cf.  Table 4), the central coordination mode appears to lead to significantly higher final performances and a significantly higher speed of performance improvement. The more complex the interdependencies among decisions are, the more advantageous the central coordination mode appears to be. From the single-objective perspective, it is striking that the achieved final performances for one objective (i.e., that objective that, according to the schism approach, was pursued in the last observation period) reaches a relatively high performance level of up to 0.9841. In the case of* short-run schism* (cf.  Table 5), similar to the long-run schism approach, the central mode of coordination appears to be significantly superior to the decentral mode. In the case of short-run schism approaches, the differences between the performances achieved with the central and the decentral coordination mode are significantly higher than the corresponding differences in the case of the long-run schism approach.

From the single-objective perspective, the achieved performances do not reach such a high level as in the case of long-run schism approaches and the differences between the final performances and the speed of performance improvement achieved with respect to the two objectives are not as high as they are in the case of long-run schism.

### 3.4. Evaluation across Multiobjective Decision Making Policies

After having outlined sensitivity of performance measures to organizational design elements separately for each multiobjective decision making policy, the following section analyses the differences in the achieved final performances and the speed of performance improvement across the different multiobjective decision making approaches.

One feature that can be observed for all multiobjective decision making approaches in combination with all investigated coordination modes is that an increase in the complexity of the interdependencies among decisions leads to a decrease in the achieved performances and to a decrease in the speed of performance improvement.

Furthermore, the presented results indicate that the multiobjective decision making approach of equal weighting and the satisficing approaches (in which the aspiration level is fixed for the objective with the less complex interactions among decisions) appear to lead to significantly higher performances than the short-run schism approach and the long-run schism-approach. However, between the performances achieved with the equal weighting approach and the performances achieved with the satisficing approach (aspiration level fixed for the objective with the less complex interactions), at best, slight differences can be observed. For the majority of setups, the aspiration level approach (in which the aspiration level is fixed for the objective with the more complex interactions among decisions) can be ranked third with respect to final performances and speed of performance improvement.

For schism approaches, whether short-run or long-run schism leads to the higher performances, appears to critically hinge on which coordination mode is utilized. For the majority of cases, the results suggest that in the central coordination mode the short-run schism approach is superior to the long-run schism approach. With decentral coordination, on the contrary, the long-run schism approach appears to lead to higher performances than the short-run schism approach. However, it has to be noted that in the case of the central coordination mode, for the majority of cases the differences between the short-run and the long-runs schism approach are marginal. In the case of the decentral coordination mode, significantly higher differences between the performances achieved with the short-run and the long-run decision making policy can be observed than in the central coordination mode. For the central as well as for the decentral coordination mode, the differences between the performances achieved with the different multiobjective decision making policies increase with increasing interdependencies among decisions.

## 4. Discussion

Burns and Stalker [45] distinguish between mechanistic and organic patterns of organizational design. One characteristic of mechanistic organizations is that they are characterized by a strict authority whereby the decision making authority is centralized. Organic organizations, on the contrary, are more open systems and the decision making is decentralized. Referring to our simulation model, mechanistic organizations correspond to the central coordination mode, while organic organizations refer to organizations that apply the decentral coordination mode. McCaskey [46] argues that mechanistic organizations are likely to achieve a higher level of performance if the organization, inter alia, performs a stable task. With respect to the coordination mode, our results do not fully support the argumentation of McCaskey [46]. Rather, we find that the choice of coordination mode only in the case of stable tasks (i.e., the method of equal weighting and the satisficing approach), at best, has a marginal influence on the achieved performance and the speed of performance improvement. Recall that the stability of the task to be performed is captured by the multiobjective decision making approach. In case of equal weighting and satisficing approaches, the organizational goal setting and, hence, the task to be performed is quite clear and stable. In case of schism approaches, on the contrary, the objective that is pursued by the organization changes in given time intervals. In particular, with an increase in the complexity of interdependencies among decisions, the decentral coordination mode even gets marginally superior to the central coordination mode. Organic organizations, on the contrary, should be advantageous for situations in which the task to be performed is not stable [46]. Our findings suggest that in the case of unstable objectives to be performed (i.e., in the case of short-run and long-run schism), the decentral coordination mode leads to significantly lower performances than the central coordination mode. Thus, our results also do not support the argumentation that organizations which are organized according to the organic approach are appropriate for setups with unstable preferences for objectives. Our results rather suggest that for the case of stable goal setting it does only make a slight difference whether the central or the decentral coordination mode is applied. For situations in which the organizational goal setting is volatile, the central coordination mode, however, appears to be significantly superior to the decentral coordination mode.

For the choice of multiobjective decision making policy, our results indicate that from the overall performance perspective relatively simple policies (i.e., equal weighting and satisficing approaches) lead to higher performances than schism approaches. It has to be noticed that for all simulated scenarios, a performance improvement can only be observed for the first few periods (approximately one third of the observation periods) while for the remaining time-steps, the performance cannot be further improved. From the single-objective perspective, our results suggest that performances per objective can, to some extent, be controlled by the coordination mode. In particular, in the case of* equal weighting*, the coordination mode appears to have an impact on whether the objective with the more complex interactions among decisions or the objective with the less complex interactions among decisions reaches a higher level of achieved performance. Thus, although in the case of equal weighting no definite preference for one of the two objectives needs to be stated, with the applied coordination mode the achieved levels of performance can, to some extent, be affected. However, it has to be noticed that there are only slight differences of the performances and the speed of performance improvement per objective achieved with the central versus the decentral coordination mode. The more complex the interdependencies among decisions with respect to the two objectives are, the higher the differences between the performances achieved on the single-objective level are.

The results indicate, at best, marginal differences of overall performance and overall speed of performance improvement achieved with satisficing approaches versus the method of equal weighting. However, from the single-objective perspective significant differences between the achieved performances can be observed. First, performance is significantly higher for the objective for which an aspriation level is stated, while the performance of the other objective cannot catch up with that performance. This means that applying aspiration levels does not solely affect the order in which objectives are pursued. In addition, the levels of performance finally achieved are crucially affected by the choice for which of the two objectives the aspiration level is fixed. Second, for satisficing approaches our results suggest that fixing the aspiration level for the objective with the less complex interdependencies among decisions leads to a higher overall performance. With respect to overall performance, this indicates to invest resources into that objective that appears to be easier to achieve (i.e., that objective with the less complex decision interdependencies). With respect to preferences for achieved performance per objective, our results give guidance for which objective to fix the aspiration level. However, with respect to organizational design our results suggest to horizontally differentiate the organization and to assign decision making authority so that the decision interdependencies with respect to the “more important" objective are less complex. This would not only lead to a higher achieved performance level with respect to that objective but also to a higher level of overall performance. However, this course of actions requires the preferences for one objective to be stable.

For the case of unstable preferences for objectives, we simulate two characterizations, that is,* the long-run schism and the short-run schism approach*. Counterintuitively, in the central coordination mode the short-run schism approach leads to slightly higher overall performances and a higher speed of performance improvement than the long-run schism approach. In the decentral coordination mode, the long-run schism approach leads to the higher levels of performance than the short-run schism approach. The more complex the interaction structure is, the more advantageous the long-run schism to the short-run schism approach is. For both characterizations of the schism policy, the central coordination mode leads to significantly higher performances. However, both schism approaches are inferior to the method of equal weighting and the satisficing approaches.

## 5. Conclusion

Our results support decision making in several ways. First, we present findings on how the horizontal differentiation and the allocation of decision making authority with respect to interdependencies among decisions contribute to the organizational performance in hierarchical organizations that pursue multiple objectives. We show that setups in which organizations are designed so that the interdependencies among decisions are minimized to intraunit interdependencies lead to significantly higher performances than setups with more complex interdependencies among decisions. Second, our results suggest that the choice of coordination mode is only of crucial relevance in case of changing preferences for objectives (i.e., schism approaches). In contrast, for stable preferences (i.e., equal weighting and satisficing approaches), at best a slight sensitivity to the coordination mode can be observed. Thus, the efficient selection of the coordination mode is of particular relevance for situations with volatile goal setting. Third, we present results on the effectivity of different multiobjective decision making policies and provide guidance on how to apply these approaches in different situations. In particular, we show that with the choice of the multiobjective decision making approach and the coordination mode, the performance of each single objective can be controlled while the overall performance remains relatively stable. Furthermore, we show that unstable preferences for objectives lead to decreasing performances. An obvious reason might be that organizations are not able to raise performance as fast as preferences change. Further simulation experiments could provide a basis for determining appropriate periods of stability.

However, our research suffers from some limitations. We limit our results to three exemplary levels of decision interdependencies with a given horizontal organizational differentiation. Furthermore, the simulated organizations pursue two objectives. Future research might investigate further natures of interdependencies among decisions. Moreover, the horizontal differentiation might be endogenized into the simulation model. Follow-up studies might also investigate setups in which organizations pursue more than two objectives at the same time. In addition to the two extreme characterizations of the coordination mode (central versus decentral), future research might investigate further mixed forms of coordination. We map an incremental search strategy whereby our agents randomly discover alternatives. Of course, the set of alternatives which the agents discover might be expanded by altering more than two bits of the decision problem at the same time. Further avenues for future research might be to map additional modes of multiobjective decision making, to investigate the impact of different incentive schemes on the performance in multiobjective setups in hierarchical organizations and to map also heterogenous multiobjective decision making approaches. Further research could also address the issue of erroneous information with regard to departmental decisions and the self-organized distribution of decision making authority among departments.

## Figures and Tables

**Table 1:** Equal weighting.

Interdependencies	Final performances			Average performances		
obj 1/obj 2	P_1_^t=100^	P_2_^t=100^	P_all_^t=100^	P_1_^avg^	P_2_^avg^	P_all_^avg^
Panel A: coordination mode: central						
Low/low	0.8984	0.8994	0.8989	0.8941	0.8949	0.8945
Low/medium	0.8777	0.8737	0.8757	0.8734	0.8694	0.8714
Medium/medium	0.8515	0.8475	0.8495	0.8479	0.8437	0.8458
Low/high	0.8515	0.8508	0.8512	0.8478	0.8474	0.8476
Medium/high	0.8215	0.8334	0.8274	0.8186	0.8303	0.8245
High/high	0.8089	0.8084	0.8087	0.8070	0.8063	0.8066

Panel B: coordination mode: decentral						
Low/low	0.8987	0.8975	0.8981	0.8957	0.8945	0.8951
Low/medium	0.9004	0.8705	0.8855	0.8967	0.8638	0.8803
Medium/medium	0.8599	0.8596	0.8598	0.8530	0.8525	0.8527
Low/high	0.8961	0.8415	0.8688	0.8909	0.8310	0.8609
Medium/high	0.8457	0.8323	0.8390	0.8369	0.8198	0.8284
High/high	0.8121	0.8153	0.8137	0.7989	0.8023	0.8006

Incentivisation: *w*_*g*_^own_*d*_^ = 1 and *w*_*g*_^res_*d*_^ = 0.5. Results are based on 450 landscapes, each with 20 adaptive walks. obj = objective, confidence intervals vary from 0.002 to 0.005 on the 99.9% level.

**Table 2:** Satisficing approach, aspiration level for objective 1: *s*_1_ = 0.8.

Interdependencies	Final performances			Average performances		
obj 1/obj 2	P_1_^t=100^	P_2_^t=100^	P_all_^t=100^	P_1_^avg^	P_2_^avg^	P_all_^avg^
Panel A: coordination mode: central						
Low/low	0.9090	0.8818	0.8954	0.9030	0.8700	0.8865
Low/medium	0.9065	0.8503	0.8784	0.9005	0.8393	0.8699
Medium/medium	0.8857	0.7849	0.8353	0.8794	0.7762	0.8278
Low/high	0.8996	0.8293	0.8645	0.8938	0.8201	0.8570
Medium/high	0.8784	0.7850	0.8317	0.8729	0.7768	0.8248
High/high	0.8568	0.7117	0.7842	0.8526	0.7073	0.7799

Panel B: coordination mode: decentral						
Low/low	0.9122	0.8783	0.8952	0.9104	0.8715	0.8910
Low/medium	0.9166	0.8505	0.8836	0.9146	0.8422	0.8784
Medium/medium	0.8907	0.7983	0.8445	0.8851	0.7889	0.8370
Low/high	0.9130	0.8259	0.8695	0.9110	0.8166	0.8638
Medium/high	0.8871	0.7888	0.8379	0.8817	0.7795	0.8306
High/high	0.8573	0.7134	0.7853	0.8482	0.7074	0.7778

Incentivisation: *w*_*g*_^own_*d*_^ = 1 and *w*_*g*_^res_*d*_^ = 0.5. Results are based on 450 landscapes, each with 20 adaptive walks. obj = objective, confidence intervals vary from 0.002 to 0.003 on the 99.9% level.

**Table 3:** Satisficing approach, aspiration level for objective 2: *s*_2_ = 0.8.

Interdependencies	Final performances			Average performances		
obj 1/obj 2	P_1_^t=100^	P_2_^t=100^	P_all_^t=100^	P_1_^avg^	P_2_^avg^	P_all_^avg^
Panel A: coordination mode: central						
Low/low	0.8814	0.9076	0.8945	0.8967	0.9016	0.8856
Low/medium	0.8197	0.8945	0.8571	0.8098	0.8877	0.8488
Medium/medium	0.7918	0.8876	0.8397	0.7826	0.8815	0.8321
Low/high	0.7338	0.8616	0.7977	0.7296	0.8569	0.7932
Medium/high	0.7146	0.8606	0.7876	0.7103	0.8561	0.7832
High/high	0.7080	0.8568	0.7824	0.7037	0.8525	0.7781

Panel B: coordination mode: decentral						
Low/low	0.8803	0.9089	0.8946	0.8734	0.9073	0.8903
Low/medium	0.8324	0.8917	0.8620	0.8226	0.8860	0.8543
Medium/medium	0.7980	0.8901	0.8441	0.7885	0.8848	0.8366
Low/high	0.7389	0.8609	0.7999	0.7328	0.8517	0.7922
Medium/high	0.7144	0.8588	0.7866	0.7084	0.8495	0.7789
High/high	0.7132	0.8587	0.7859	0.7070	0.8495	0.7783

Incentivisation: *w*_*g*_^own_*d*_^ = 1 and *w*_*g*_^res_*d*_^ = 0.5. Results are based on 450 landscapes, each with 20 adaptive walks. obj = objective, confidence intervals vary from 0.001 to 0.004 on the 99.9% level.

**Table 4:** Long-run schism.

Interdependencies	Final performances			Average performances		
obj 1/obj 2	*P*_1_^*t*=100^	*P*_2_^*t*=100^	*P*_all_^*t*=100^	*P*_1_^avg^	*P*_2_^avg^	*P*_all_^avg^
Panel A: coordination mode: central						
Low/low	0.9795	0.6903	0.8349	0.8262	0.8335	0.8299
Low/medium	0.9831	0.6613	0.8222	0.8527	0.7859	0.8193
Medium/medium	0.9289	0.7968	0.8178	0.7990	0.8039	0.8014
Low/high	0.9841	0.6479	0.8160	0.8777	0.7516	0.8147
Medium/high	0.9358	0.6743	0.8050	0.8182	0.7617	0.7899
High/high	0.8748	0.6926	0.7837	0.7652	0.7668	0.7660

Panel B: coordination mode: decentral						
Low/low	0.9826	0.6864	0.8345	0.8270	0.8266	0.8268
Low/medium	0.9812	0.6478	0.8145	0.8279	0.7596	0.7938
Medium/medium	0.9206	0.6666	0.7936	0.7673	0.7686	0.7680
Low/high	0.9841	0.6455	0.8148	0.8345	0.7080	0.7712
Medium/high	0.9231	0.6566	0.7899	0.7726	0.7115	0.7420
High/high	0.8261	0.6711	0.7486	0.7148	0.7184	0.7166

Incentivisation: *r*_*z*_^own_*d*_^ = 1 and *r*_*z*_^res_*d*_^ = 0.5. Results are based on 450 landscapes, each with 20 adaptive walks. obj = objective, confidence intervals vary from 0.001 to 0.004 on the 99.9% level.

**Table 5:** Short-run schism.

Interdependencies	Final performances			Average performances		
obj 1/obj 2	P_1_^t=100^	P_2_^t=100^	P_all_^t=100^	P_1_^avg^	P_2_^avg^	P_all_^avg^
Panel A: coordination mode: central						
Low/low	0.8878	0.8093	0.8486	0.8434	0.8429	0.8432
Low/medium	0.9046	0.7465	0.8256	0.8624	0.7897	0.8261
Medium/medium	0.8579	0.7790	0.8185	0.8062	0.8081	0.8072
Low/high	0.9150	0.6954	0.8052	0.8751	0.7515	0.8133
Medium/high	0.8664	0.7373	0.8018	0.8196	0.7683	0.7940
High/high	0.8382	0.7711	0.8047	0.7821	0.7823	0.7822

Panel B: coordination mode: decentral						
Low/low	0.8859	0.7638	0.8248	0.8210	0.8246	0.8228
Low/medium	0.8842	0.7055	0.7949	0.8177	0.7402	0.7790
Medium/medium	0.7748	0.6979	0.7364	0.7311	0.7317	0.7314
Low/high	0.8865	0.6766	0.7816	0.8164	0.6875	0.7520
Medium/high	0.7750	0.6672	0.7211	0.7308	0.6788	0.7048
High/high	0.7133	0.6772	0.6953	0.6841	0.6846	0.6843

Incentivisation: *r*_*z*_^own_*d*_^ = 1 and *r*_*z*_^res_*d*_^ = 1. Results are based on 450 landscapes, each with 20 adaptive walks. obj = objective, confidence intervals vary from 0.002 to 0.005 on the 99.9% level.
